# Adolescents’ difficulties, strengths and feelings of insecurity: a cross-sectional descriptive survey in Finland

**DOI:** 10.1007/s44192-023-00043-4

**Published:** 2023-08-31

**Authors:** Pinja Kokkonen, Christina Athanasopoulou, Helena Leino-Kilpi, Pauli Puukka, Evanthia Sakellari

**Affiliations:** 1 Department of Nursing Science, University of Turku, Turku, Finland; 2 Department of Occupational Therapy, University of West Attica, Athens, Greece; 3 Turku University Hospital, Turku, Finland; 4 University of Turku, Turku, Finland; 5 Department of Public and Community Health, University of West Attica, Athens, Greece; 6 196 Alexandras Av., 11521 Athens, Greece

**Keywords:** Adolescents, Youth, Difficulty, Strength, Insecurity, Finland, SDQ

## Abstract

The present study aimed to describe adolescents’ self-reported emotional and behavioural strengths and difficulties, as well as their insecurity feeling. In addition, the aim was to examine the association with background characteristics, and explore the association between strengths and difficulties and insecurity factors. The study was conducted among 114 secondary school pupils in Finland, using an online questionnaire. Adolescents’ emotional and behavioural difficulties and strengths, were mostly classified as normal. Strengths and Difficulties Questionnaire total score as well as internal and external score, were inversely associated with insecurity factors. Girls had significantly higher prosocial behavior compared to boys (P = 0.0007). The age of adolescents was found to be related to their internal difficulties (P = 0.02) and prosocial behavior (P = 0.01). Adolescent’s perception of their family relations as poor was associated with external difficulties (P = 0.04). The current results, can be helpful for mental health professionals who work with adolescents in order to implement appropriate and needs specific mental health promotion interventions at individual but also community level. Finally, more research is needed to validate measures for insecurity. This will support mental health professionals in their clinical practice by providing them with all the important factors needed to support adolescents.

## Introduction

Out of the 7.2 billion people worldwide, 3 billion are younger than 25 years, which represents 42% of the world population [1]. Although our world has more young people than ever before, this age group commonly faces mental health problems such as depression, which is one of the leading causes of illness and disability [2]. Moreover, mental health problems during childhood have long-term effects, such as fewer years of education and about 20% lower level of adult family income [3] It is also important to notice that studies have supported the mental health problems issues raised among adolescents during the COVID-19 pandemic. It has been found an increase in adolescent depressive symptoms [4, 5] and poorer mental wellbeing [5] as well as a decrease in life satisfaction [4]. According to the W.H.O. [2], the consequences of not addressing adolescent mental health conditions can extend to adulthood, impairing physical and mental health, while minimizing opportunities to lead fulfilling lives as adults. Thus, it is imperative to focus on adolescents’ mental health promotion and acknowledge that adolescence is a vital stage of life for identification and prevention of mental health problems [6].

However, mental health needs of young people are still unmet, even in high-income countries, while at the policy level, adolescents and their concerns should be at the focus in rapidly changing societies [7]. Europe has undergone significant changes that may affect citizens’ security. Since 2008, Europe has faced a financial crisis [8], and some of the major cities have been targeted by terrorists. Studies indicate that financial issues have become a new source of concern among adolescents [9]. Additionally, the number of refugees, including young people, has been on the rise. Certain young people find themselves vulnerable to particular risks in displacement situations, such as separation from family, disability, or becoming victims of sexual and gender-based violence [10]. Any of insecurity may negatively impact people’s present and future lives. Financial insecurity is a significant concern among young people, while older people tend to feel the most physical insecurity across all age groups [11]. Research suggests that adolescents experiencing feelings of insecurity tend to be quiet, introverted, withdrawn, nervous and prone to anxiety [12]. These adolescents are typically of normal intelligence, well-behaved, and do not cause trouble for their parents, but are internally disturbed and anxious about what may happen to them. Although the Finnish people’s sense of insecurity has decreased since 2015, the number of people experiencing insecurity has increased [13]. It has been found that the girls were found more insecure than the boys [12, 14], the adolescents of nuclear families were found more insecure than the adolescents belonging to bigger families [12] and adolescents of low socio-economic status were more insecure than those of the high socio-economic status [14]. In addition, young people tend to see the future as more secure when compared to adults and older individual [13].

It is supported that there are several factors associated with mental health in adolescents’ daily living [15]. One such factor is the family's socio-economic situation (SES). Low SES is related to higher levels of mental health difficulties, loneliness, and low self-esteem among [16, 17]. Additionally, according to Torikka et al. [18] depression nearly doubled among Finnish adolescents with unemployed and low-educated parents between 2000 and 2011. In addition, it has been found that adolescents from high-affluence families report higher levels of life satisfaction and excellent health, and lower levels of multiple health complaints [19]. Family relations’ impact to children’s mental health has been as well highlighted in the literature. Parental care which is a parent–child relationship dimension and it reflects a continuum from affectionate, warm, responsive parenting to cold and unresponsive parenting is related to mental well-being with higher parental care being related to higher mental well-being even in the adulthood [20]. Moreover, secure attachment style is associated in decreased level of children’s emotional and behavioural difficulties and high pro-social behaviour [21]. Poor parental relations are connected to the mental health difficulties [17]. Spending time together as a family and having a positive atmosphere at home are related to adolescents’ satisfaction to their body appearance [22], while, experiences of neglect and antipathy in a childhood are connected to the anxiety in adolescence [23]. On the other hand, protective and supportive factors for mental health are high activity in hobbies and jobs, high amount of social contacts and a proper school performance as well as problem solving skills, social support, supportive care-giving, getting along with peers are recognized to support mental health among adolescents [24].

Insecurity is seen as the lack of basic security and balance on a social (parents, relatives, friends), external (international relationships and global circumstances), and/or societal level (security resources, social support, secure environment) [25]. There have been studies examining the feelings of insecurity among young people [12, 26]. Adolescents themselves have connected the insecurity to their inner feelings and emotions, and also to their social relationships. In addition, they have defined external reality, like socio-economic ill-being, violence and war as one aspect of insecurity [24]. Climate crisis affects all domains of adolescent wellbeing, feeling hopeless and helpless [27]. Young people are considered to be at greater vulnerability to the negative effects of climate change by virtue of their developing coping capacity [28]. An earlier study found that family relationships and in particular those with parents, are the key elements for security, while relationships with friends and other social learning environments such as school, media and the internet are also part of the growing environment, and they important contexts of security but also sources of insecurity [29].

To our knowledge, so far there is no study that has investigated adolescents’ insecurity and their relation to their emotional difficulties. Therefore, the purpose of the study was to describe adolescents’ self-reported emotional and behavioural strengths and difficulties, as well as their insecurity feelings. In addition, the aim was to examine strengths and difficulties in relation to adolescents’ background characteristics, and finally explore the association between strengths and difficulties and insecurity factors. Proper questionnaires have been identified in order to assess all these and they are described in the methods section. The ultimate goal was to provide useful information about adolescents’ perceptions of their emotional and behavioural difficulties, strengths, and feelings of insecurity, in order for mental health professionals to implement appropriate interventions according to adolescents’ needs.

## Methods and setting

To address the research questions, a cross-sectional and descriptive survey design was used. The data was collected from one randomly selected public secondary school in Western Finland. All pupils (225) in the school were invited to the study. There are approximately 175,330 secondary school pupils in Finland, with about 14,530 pupils in the specific region [30]. Ιt was calculated that with the sample size of 110 or more participants the study would have 95% power to detect significant associations in a model, with an effect size of 0.2 or more and at a significance level of 0.05. The significance level was set at 0.05 or 5% as the most usual and international proportion. The inclusion criteria for this study were: age between 12 and 17 years, understanding of Finnish language, and consent from parents/guardians as well as the pupils themselves. Data was collected, during autumn 2016 via an online questionnaire, which took approximately 20 min to fill in, during school time facilitated by the teachers. The questionnaire was self-reported in order to avoid socially desirable answers from the participants. It included questions of background participants’ characteristics and two instruments that are described below.

### Instruments and measures

For the data collection, the online questionnaire included the Finnish version [31] of Goodman’s the Strengths and Difficulties Questionnaire (SDQ) [32]. The SDQ consists of 25 items, measuring 25 attributes divided in five scales: (1) emotional symptoms (5 items), (2) conduct problems (5 items), (3) hyperactivity/inattention (5 items), (4) peer relationship problems (5 items), and (5) prosocial behaviour (5 items). Each item is scored on a 3-point Likert scale, ranging from 0 “Not true” to 2 “Certainly true”. Twenty of the items are scored summative to provide a total difficulties score (score range 0–40). The SDQ scores classified answers: (1) “normal” for those scored between 0–15; (2) “borderline” for those scored between 16–19, and (3) “abnormal” for those scored between 20 and 40 (SDQ-info, 2017). Higher summative scores indicate larger problems in four problem areas and higher positive behaviour in pro-social behaviour subscale [32]. For normal prosocial behaviour, recommended values are six or over and scoring five is interpreted as borderline difficulties [33]. According to Goodman et al. [34], when conducting a study in a low-risk or general population sample—as in this study—three subscales can be used: (1) internalizing (emotional and peer relationship problems), (2) externalizing (conduct and hyperactivity problems) and (3) prosocial scale. Thus, in this study, these subscales were selected because the sample composed of general population adolescents. SDQ has been used in both clinical and community samples [29]. It has shown good psychometric properties and internal consistency in earlier Finnish study focused on children aged 7–15 [25]. SDQ total difficulties scores across Nordic countries have shown to be quite similar. For example, in Finland total difficulties mean score has been 11.4 and in Norway between 9.8 and 11.1 [35].

Furthermore, feelings of insecurity, were assessed based on the Youth Barometer questionnaire by Myllyniemi [22] which consists of 37 items, measuring 6 insecurity factors: (1) “Being a victim” (7 items concerning security of living area, physical and mental and sexual violence towards self, being lonely, being discriminated and being healthy); (2) “Unethical” (9 items connected to uncertainty and risks created by human action globally); (3) “Polarization and losing a joint responsibility” (5 items concerning the inequality, the weakening of sense of community, an aging of our population, a racist violence and youth discrimination); (4) “International relations and religions” (8 items about Russia being so close to Finland, Islam, an expanding of EU, globalisation of economics, population growth, conflicts between religions, increase of immigrants and weapons of mass destruction); (5) “Catastrophes” (3 items about different kinds of environmental catastrophes, but a militarily attack to Finland belongs to this factor too); (6) “Personal threats” (5 items involving insecurity concerning own incomes, own security, getting employed, security of family members and studying) [26]. Participants rated each item on a 5-point Likert scale from 1 “very little or not at all” to 5 “very much” (an option of “cannot answer” was included separate from the scale) [26]. Youth Barometer is an annual telephone survey (since 1994) focusing on young people aged 15 to 29 living in Finland. The research methods used in the Youth Barometer study were explorative factor analysis, mean value studies and MCA analysis [36].

In addition, participants’ background characteristics (such as gender and age) were recorded, and the family’s socio-economic situation was assessed by asking about the father’s employment status, mother’s employment status, father’s educational level, mother’s educational level, perceived family finances, perceived welfare of the living area, living conditions, and perceived family relations.The assessment of family relations was on a scale 10 to 1, where 10 was (“we have very good relationships in our family”) to 1 (“We have very bad relationships in our family”). In the current study, Cronbach’s alpha exceeded 0.70 which indicates an acceptable reliability.

### Ethical considerations

This research was performed in line with the principles of the Declaration of Helsinki. Approval was granted by the Ethics Committee of Turku University (Number 29/2016). The school principal granted permission for the study to be conducted [37]. Potential participants and their parents/guardians were informed about the study with a separate letter in which anonymity and voluntariness were emphasized. All participants and their parents/guardians provided written informed consent. Even if pupils had their parents’/guardians’ permission to take part in the study, their participation was still their own choice.

### Data analysis

Data was analysed using the SAS 9.4 software (SAS Institute Inc., Cary, North Carolina, USA). There were no missing data. Descriptive statistics such as frequencies, percentages, means, medians (MD), and standard deviations (SD) were used to describe the background variables, SDQ scores, and factors of insecurity. As the data for SDQ scores were normally distributed, parametric tests were selected. Differences in the SDQ scores by gender and parents’ employment status were tested by T-tests. Differences in the SDQ scores by background variables with more than two categories were tested with the analysis of variance. Due to skewed distributions factors of insecurity were compared with each other by nonparametric Wilcoxon matched-pairs signed-ranks test. Spearman correlation was used to test the correlation between SDQ scores and factors of insecurity. Cohen’s effect sizes were used. Effect sizes less than 0.3 were considered small, from 0.3 to 0.5 medium and over 0.5 large. A significance level of 0.05 was used for all tests.

## Results

### Participants’ characteristics

In total, 114 pupils participated in the study. Αll pupils aged 12–17 years were invited to participate in the study and a response rate of 50.7 was achieved which is acceptable [38]. The majority of the sample, was female (n = 63, 55.3%), aged between 15 and 17 years (n = 57), lived with both parents (n = 72), had an employed father (n = 90) and/or mother (n = 94), did not know the father’s educational level (n = 47), mother’s education was at a university level (n = 53), the perceived family’s financial situation was mostly very good (n = 43) or quite good (n = 43). The perceived welfare of the living area was mostly very good (n = 58), with very good family relations (n = 76) (Table 1).

**Table 1:** Participants’ characteristics

	N	%
Gender		
Female	63	56.3
Male	49	43.8
Age		
11–13	36	32.7
14	17	15.5
15–17	57	51.8
Father’s employment status		
Yes	90	78.9
Other	24	21.1
Mother’s employment status		
Yes	94	82.5
Other	20	17.5
Father’s educational level		
University	40	35.1
I don’t know	47	41.2
Basic/practical	27	23.7
Mother’s educational level		
University	53	46.5
I don’t know	41	36.0
Basic/practical	20	17.5
Perceived family financial situation		
Very good	43	37.7
Quite good	43	37.7
Average	16	14.0
Quite poor	12	10.5
Perceived welfare of the living area		
Quite good	39	34.2
Very good	58	50.9
Quite poor	17	14.9
Living condition		
Both parents	72	63.2
One parent	34	29.8
Other	8	7.0
Perceived family relations		
1–5	14	12.3
6–8	24	21.1
9–10	76	66.7

### Emotional and behavioural difficulties and strengths

Most of the mean scores in total difficulties and in sub-scales were classified as “normal” having total mean scores under 15, and internal and external mean scores under 10 [33] (Table 1). However, some groups’ values were a bit over the normal score, classifying them in the borderline group. For example, adolescents who rated their family relations with the weakest score (15.93, SD 5.03), and/or who rated their family’s financial situation quite poor (15.58, SD 7.23), both scored over 15 in total mean scores.

Most of the mean scores in prosocial behaviour, were classified as “normal” having values six or above. There were some groups who had mean scores a bit below of six, but not five which is interpreted as borderline difficulties [27]. The groups with prosocial mean scores slightly under six were those: aged 14 years (mean 5.3, SD 3.1), who perceived their family financial situation quite poor (mean 5.8, SD 2.52), who received their family relations the poorest (mean 5.7, SD 2.84).

### Feelings of insecurity

The insecurity factor with the highest mean score was “Being a victim”, with mean score 4.3 out of maximum 5 (“Very much”) (SD 0.96, MD 4.71). This factor was significantly higher than all other factors of insecurity (P < 0.001 for total, internal and external scores). There were no other significant statistical differences between the other five insecurity factors. The factor “Personal threats” (such as insecurity concerning own income and security, getting employed, security of family members and studying) had a mean score of 3.84 (SD 1.06, MD 4.00). As a third insecurity factor, was found to be “Polarization” in our society (such as weakening of communal collaboration, racist violence and youth discrimination) with mean score 3.75 (SD 1.23, MD 4.00). The fourth insecurity factor “International relations” (such as globalization of economics, conflicts between religions and global population growth were in a forth causing insecurity) had a mean score of 3.72 (SD 1.12, MD 4.00), followed by “Unethicality” (such as factors caused by people’s unethical action) with mean score 3.67 (SD 1.14, MD 4.00), and “Catastrophes” (such as different kinds of environmental involving military attack to Finland) with mean score 3.54 (SD 1.32, MD 4.00).

### Association with sociodemographic characteristics

The results exhibit some statistically significant associations between adolescents’ background characteristics and their strengths and difficulties (Table 2). More specific, girls had significantly higher score on prosocial behavior as compared to boys (P = 0.0007, effect size = 0.70). Higher score on internal difficulties was found in ages 11 to 13 old (P = 0.02, effect size = 0.27), while score on the prosocial behaviour was also higher in ages 11 to 13 old (P = 0.01, effect size = 0.26). Living with both or only one parent or with somebody else was related to total difficulties (P = 0.02, effect size = 0.20), but also to external (P = 0.03, effect size = 0.23) and internal difficulties (P = 0.048, effect size = 0.20). Adolescent’s poor perception of their family relations was associated with external difficulties (P = 0.04, effect size = 0.24).

**Table 2:** Strengths and difficulties (SDQ) and correlations with background characteristics

Background characteristics	SDQ total	P	SDQ external	P	SDQ internal	P	SDQ prosocial	P
	Mean (SD)		Mean (SD)		Mean (SD)		Mean (SD)	
All (n = 114)	12.18 (7.39)		5.96 (4.00)		6.22 (4.17)		6.81 (2.30)	
Gender								
Female (n = 63)	11.98 (6.81)	0.9333	5.97 (3.98)	0.8852	6.02 (3.79)	0.7714	7.46 (1.79)	0.0007**
Male (n = 49)	12.10 (8.06)		5.86 (4.10)		6.24 (4.53)		5.92 (2.61)	
Age								
11–13 (n = 36)	14.06 (9.09)	0.0962	6.83 (4.15)	0.1560	7.25 (5.36)	0.0201*	7.14 (2.21)	0.0137*
14 (n = 17)	10.06 (5.43)		6.12 (3.59)		3.94 (2.77)		5.30 (3.10)	
15–17 (n = 57)	11.28 (6.16)		5.21 (3.94)		6.07 (3.12)		7.00 (1.95)	
Father’s employment status								
Yes (n = 90)	12.22 (7.54)	0.8966	5.91 (4.11)	0.7822	6.32 (4.20)	0.6117	6.84 (2.23)	0.7377
Other (n = 24)	12.00 (6.97)		6.17 (3.63)		5.83 (4.09)		6.67 (2.56)	
Mother’s employment status								
Yes (n = 94)	12.01 (7.38)	0.6079	5.99 (4.02)	0.8882	6.03 (4.13)	0.299	6.76 (2.39)	0.6044
Other (n = 20)	12.95 (7.58)		5.85 (4.00)		7.10 (4.32)		7.05 (1.85)	
Father’s educational level								
University (n = 40)	11.1 (7.06)	0.4827	5.00 (4.10)	0.4827	6.13 (3.49)	0.6201	6.95 (1.83)	0.2451
I don’t know (n = 47)	12.4 (7.24)		6.57 (3.53)		5.91 (4.42)		6.40 (2.82)	
Basic/practical (n = 27)	13.22 (8.17)		6.33 (4.46)		6.88 (4.67)		7.30 (1.79)	
Mother’s educational level								
University (n = 53)	11.30 (7.36)	0.4913	5.04 (3.90)	0.0681	6.28 (3.98)	0.9433	7.28 (2.05)	0.1119
I don’t know (n = 41)	12.78 (7.97)		6.73 (3.65)		6.05 (4.86)		6.46 (2.59)	
Basic/practical (n = 20)	13.25 (6.21)		6.85 (4.57)		6.40 (3.16)		6.25 (2.12)	
Perceived family financial situation								
Very good (n = 43)	10.60 (7.45)	0.1700	5.35 (4.13)	0.1478	5.26 (4.07)	0.2420	6.60 (2.54)	0.1102
Quite good (n = 43)	12.95 (7.65)		6.35 (3.98)		6.63 (4.28)		6.88 (2.11)	
Average (n = 16)	11.75 (5.93)		5.06 (3.26)		6.69 (3.66)		7.88 (1.59)	
Quite poor (n = 12)	15.58 (7.23)		8.00 (4.07)		7.58 (4.50)		5.83 (2.52)	
Perceived welfare of the living area								
Quite good (n = 39)	12.21 (6.83)	0.9945	5.79 (3.76)	0.8137	6.41 (3.87)	0.7567	6.51 (2.29)	0.3764
Very good (n = 58)	12.21 (8.39)		5.91 (4.32)		6.29 (4.68)		7.10 (2.23)	
Quite poor (n = 17)	12.00 (4.91)		6.53 (3.52)		5.53 (2.92)		6.47 (2.53)	
Living condition								
Both parents (n = 72)	10.74 (6.20)	0.0231*	5.24 (3.67)	0.0354*	5.50 (3.32)	0.0480*	6.70 (2.26)	0.6295
One parent (n = 34)	14.68 (8.51)		7.12 (4.18)		7.59 (5.15)		7.12 (2.24)	
Other (n = 8)	14.50 (9.65)		7.63 (4.87)		6.88 (5.36)		6.50 (2.98)	
Perceived family relations								
1–5 (n = 14)	15.93 (5.03)	0.1276	8.36 (3.46)	0.0488*	7.64 (4.07)	0.3548	5.71 (2.84)	0.1068
6–8 (n = 24)	11.67 (6.91)		6.00 (3.97)		5.67 (3.64)		6.58 (2.40)	
9–10 (n = 76)	11.64 (7.76)		5.51 (3.99)		6.13 (4.33)		7.08 (2.12)	

^*^ P < 0.05, ** P < 0.001

More specific, girls had significantly higher prosocial behavior compared to boys (P = 0.0007). Adolescents’ age was related to their internal difficulties (P = 0.02) and to the prosocial behavior (P = 0.01). Living with both or only one parent or with somebody else was related to total difficulties (P = 0.02), but also to external (P = 0.03) and internal difficulties (P = 0.048). Adolescent’s perception of their family relations as poor was associated with external difficulties (P = 0.04).

SDQ total score, and internal and external score, were inversely associated with insecurity of “Being a victim” followed by “Personal threats” (Table 3). The same pattern of association was seen in other factors of insecurity, as well, but only six of them reached statistical significance. The prosocial score did not correlate significantly with any factor of insecurity.

**Table 3:** Correlations between Insecurities and Strengths and difficulties (SDQ)

Insecurities	SDQ total	SDQ external	SDQ internal	SDQ prosocial
Being a victim	− 0.452***	− 0.364***	− 0.396**	0.186
Unethicality	− 0.216*	− 0.113	− 0.263*	− 0.158
Polarisation	− 0.233*	− 0.167	− 0.227*	− 0.118
International relations	− 0.224*	− 0.157	− 0.241*	− 0.075
Catastrophes	− 0.177	− 0.119	− 0.189	− 0.177
Personal threats	− 0.392***	− 0.280**	− 0.383***	0.061

^*^ P < 0.05, ** P < 0.01, *** P < 0.001

## Discussion

The current study aimed to explore adolescents’ emotional and behavioural strengths and difficulties, and their association with the factors creating insecurity, in order to support adolescents’ mental health promotion within the context of insecurity. A novel finding of our study was the inverse association between adolescents’ difficulties and feelings of insecurity, when difficulties decreased the amount feeling of insecurity raised. Insecurity related to personal threats and being a victim was associated with emotional and behavioral difficulties decreased. The same pattern emerged with other insecurity factors as well, although the correlation was not significant. “Being a victim” was the factor that created the most insecurity. Emotional and behavioural difficulties were also related to adolescents’ age, living conditions and family relations.

Gender differences in prosocial behavior were found, with earlier studies supporting the finding that girls tend to exhibit more prosocial behavior than boys [21, 39–41]. However, no gender differences were observed in emotional and behavioral difficulties. This suggests that the strength of girls in prosocial behavior does not afford them better protection against such difficulties compared to boys. It is worth discussing what prosocial behavior means to girls. Previous studies have shown that boys experience more peer problems than girls [21]. For example, among Finnish secondary school pupils, 12% of boys and half of girls (6%) report they do not have any close friends [42]. However, research shows that male adolescents tend to have multiple friendships and spend more time with friends than females do. Boys also engage in more activities and exercise with their friends, while girls tend to prefer personal communication in their friendships [19, 43]. Results concerning gender differences in peer relations seems to be two-fold. According to Goodman et al. [34], SDQ’s prosocial behaviour measures consideration, sharing, caring, being kind to kids and helping others. It is interesting to consider social biases when examining the higher levels of prosocial behavior reported by females. Are these results a true reflection of reality, or are girls simply providing answers they believe are socially expected of them. Earlier evidence suggests that adolescents perceive different societal expectations for boys and girls [44]. One explanation for girls’ higher prosocial behaviour could be that adolescent females and males have different ways of being social [45]. The SDQ’s prosocial behaviour scale may target the behaviours that are more commonly exhibited by girls [46].

Moreover, living conditions were shown to be related to adolescents’ mental health difficulties. For example, adolescents residing with both parents had lower difficulties compared to those living with only one parent or those in other situations. Previous studies have also indicated that single-parent households are associated with more emotional and behavioral problems and increased use of mental health services by young people [47, 48]. However, Nilsen et al. [49] noticed that adolescents living in joint physical custody do not have more difficulties than their peers residing with both parents. Similarly, living in a patchwork family is not associated with higher difficulties [47]. The sample of the current study did not made possible to compare different kind of family combinations accurately in order to confirm or not similar findings from previous studies.

In the current study, the adolescents who perceived their family's financial situation the poorest, had slightly higher total difficulties of the normal level. Varga et al. [16] found that adolescents’ own perception of family’s financial situation is a more powerful factor to mental health than an objective measure of family’s financial status, such as parents’ educational background.

Those adolescents who had fewer difficulties, felt more insecure about various factors compared to those who had more difficulties. A previous study found that 59% of Finns believe that we will be living in more insecure world in the next 5 years [13]. Recent literature suggests that children’s emotional reactivity is related to worrying, and intolerance of uncertainty mediates this relationship [50] while worrying and intolerance of uncertainty are associated with anxiety and other emotional difficulties [51, 52]. SDQ scores were inversely associated with insecurity factors. Although we did not examine intolerance of uncertainty, which could have explained our findings, the results were still unexpected. One way to consider this relation between feeling of insecurity and emotional difficulties is to broaden the perspective. In social sciences, both security and insecurity as concepts can describe one’s communication/orientation to the society and to the world [35]. If this perspective is applied to our study, adolescents, who have many difficulties with their mental health, may not be as orientated to the world changing around them because of their own difficulties. Conversely, awareness of the world and the insecurity that stems from it may not necessarily cause emotional or behavioral difficulties in adolescents. In fact, difficulties could strengthen adolescents' internal values [53]. However, insecurity is a complex concept, affected by various internal and/or external factors, as discussed earlier. In a constantly changing world, feeling insecure can be considered a natural response and not necessarily negative. Mental health professionals should keep this in mind when treating adolescents, being aware of each person's unique circumstances, and remembering that the content of insecurity may differ at varying times [25].

There are several limitations to the present study. The study was conducted in one area in southwest Finland and the sample size was small and therefore, the results may restrict generalizability. The construct validity could have been strengthened by validating the SDQ against mental health instruments with age-appropriate questionnaires. Ηowever, this was beyond the scope of the study. Despite that one of the strengths of the SDQ is the opportunity of having multi-informant responses, the present study is limited to adolescents’ subjective perceptions. Whether parental and teacher reports show the same factor structure for this age group or not, needs further investigation. High scores on the SDQ have previously been associated with nonresponse in investigations [54], it could be possible that participants with lower scores are overrepresented in the current study. Similarly, it can be possible that adolescents with more severe mental health problems, would not have been part of the study as they are more likely to have been absent from school because of their difficulties [55]. Another limitation is that the current study assessed the adolescents’ perceptions about their family’s financial status, living condition, family relations and therefore their answers might be not objective. Finally, as the study is cross-sectional, it does not determine the directions of the associations.

To conclude, our study provided important insights about adolescents’ emotional and behavioural difficulties, strengths and insecurities. The results showed that higher emotional and behavioural difficulties were not associated with higher feelings of insecurity. This is an important finding for the mental health professionals who work with adolescents facing difficulties. Additionally, it is for the mental health professionals to acknowledge that adolescents with their own difficulties may perceive insecurity in a different way compared to their peers. Furthermore, very low orientation to the world, due to one’s own difficulties, could even affect on discrimination issues among adolescents. Hence, the study findings draw the attention to mental health professionals who work with adolescents to implement appropriate and needs specific mental health promotion interventions at individual but also community level. Mental health professionals should focus on mental health promotion interventions which increase feelings of security and in particular develop life skills in order for young people to face everyday insecurities, cope with stressors and enhance self-awareness and self-confidence. Finally, further research is necessary to validate measures of insecurity at, personal, social, societal, and global levels. This will support the clinical practice of mental health professionals by providing them with all the essential factors needed to support adolescents. Measures of assessing adolescents’ perception, on the other hand, would also help in regards to provide information in a salutogenic approach and assess the potentials and the internal and external resources that support security among young people.

## Data Availability

The datasets generated and/or analysed during the current study are not publicly available due to the restrictions by the ethics approval of the study.

## References

[1] W.H.O. Coming of age: adolescent health; 2018. http://www.who.int/health-topics/adolescents/coming-of-age-adolescent-health. Accessed 07 Jan 2023.

[2] W.H.O. Adolescent mental health; 2018. http://www.who.int/news-room/fact-sheets/detail/adolescent-mental-health. Accessed 07 Jan 2023.

[3] Smith JP, Smith GC (2010). Long-term economic costs of psychological problems during childhood. Soc Sci Med.

[4] Thorisdottir IE, Agustsson G, Oskarsdottir SY, Kristjansson AL, Asgeirsdottir BB, Sigfusdottir ID, Valdimarsdottir HB, Allegrante JP, Halldorsdottir T (2023). Effect of the COVID-19 pandemic on adolescent mental health and substance use up to March, 2022, in Iceland: a repeated, cross-sectional, population-based study. Lancet Child Adolesc Health.

[5] Mansfield R, Santos J, Deighton J, Hayes D, Velikonja T, Boehnke JR, Patalay P (2022). The impact of the COVID-19 pandemic on adolescent mental health: a natural experiment. R Soc Open Sci.

[6] W.H.O. Comprehensive Mental Health Action Plan; 2013. http://www.who.int/entity/mental_health/publications/action_plan/en/index.html. Accessed 07 Jan 2023.

[7] Patel V, Flisher AJ, Hetrick S, McGorry P (2007). Mental health of young people: a global public-health challenge. Lancet.

[8] Clench-Aas J, Holte A (2018). Measures that increase social equality are effective in improving life satisfaction in times of economic crisis. BMC Public.

[9] Matos MG (2016). Worries, coping strategies and well-being in adolescence: highlights from HBSC study in Portugal. Vulnerable Child Youth Stud.

[10] UNHCR (2013). UNHCR’s engagement with displaced youth.

[11] Eurostat (2015). Quality of life. Facts and views. Eurostat. Statistical books. 2015 editions.

[12] Raina S, Bhan KS (2013). A study of security-insecurity feelings among adolescents in relation to sex, family system and ordinal position. Int J Educ Plan Admin.

[13] MTS. Suomalaisten mielipiteitä ulko- ja turvallisuuspolitiikasta, maanpuolustuksesta ja turvallisuudesta. Maanpuolustuksen suunnittelukunta. Tiedotteita ja katsauksia. [Finnish opinions on foreign and security policy, national defense and security]; 2017. http://julkaisut.valtioneuvosto.fi/bitstream/handle/10024/160343/MTS%202017%20suomi%20NETTI_u.pdf?sequence=1&isAllowed=y. Accessed 07 Jan 2023.

[14] Shahana A, Asiya A (2014). A study of security-insecurity feelings among adolescents in relation to gender and socio-economic status. Indian J Psychol Sci.

[15] Das JK (2016). Interventions for adolescent mental health: an overview of systematic reviews. J Adolesc Health.

[16] Varga S, Piko BF, Fitzpatrick KM (2014). Socioeconomic inequalities in mental wellbeing among Hungarian adolescents: a cross-sectional study. Int J Equity Health.

[17] Ravens-Sieberer U, Erhart M, Gosch A, Wille N, The European KIDSCREEN Group (2008). Mental Health of children and adolescents in 12 European countries—results from the European KIDSCREEN study. Clin Psychol Psychother.

[18] Torikka A (2014). Self-reported depression is increasing among socio-economically disadvantaged adolescents—repeated cross-sectional surveys from Finland from 2000 to 2011. BMC Public Health.

[19] Inchley J et al. editors. Spotlight on adolescent health and well-being. Findings from the 2017/2018 Health Behaviour in School-aged Children (HBSC) survey in Europe and Canada. International report. Volume 1. Key findings. Copenhagen: WHO Regional Office for Europe; 2020. https://apps.who.int/iris/bitstream/handle/10665/332091/9789289055000-eng.pdf. Accessed 06 July 2023.

[20] Stafford M, Kuh DL, Gale CR, Mishra G, Richards M (2016). Parent-child relationships and offspring's positive mental wellbeing from adolescence to early older age. J Posit Psychol.

[21] Keskin G, Cam O (2010). Adolescents’ strengths and difficulties: approach to attachment styles. J Psychiatr Ment Health Nurs.

[22] Myllyniemi, S. Ed. Puolustuskannalla. Nuorisobarometri 2010 [The Youth Barometer 2010]. Opetus- ja kulttuuriministeriö. Nuorisotutkimusverkosto. Nuorisoasiain neuvottelukunta; 2010. https://tietoanuorista.fi/wp-content/uploads/2013/05/nuorisobarometri2010.pdf. Accessed 07 Jan 2023.

[23] Schimmenti A, Bifulco A (2015). Linking lack of care in childhood to anxiety disorders in emerging adulthood: the role of attachment styles. Child Adolesc Ment Health.

[24] Erol N, Simsek Z, Műnir K (2010). Mental health of adolescents reared in institutional care in Turkey. Eur Child Adolesc Psychiatry.

[25] Vornanen R, Törrönen M, Niemelä P, Miettinen J, López-Varela A (2012). The conceptualising of insecurity from the perspective of young people. Social sciences and cultural studies—: issues of language, public opinion, education and welfare.

[26] Vornanen R, Miettinen J. Nuorten turvattomuus ja epävarmuus vuonna 2010. In: Puolutuskannalla. Nuorisobarometri 2010. [Youth experiences of insecurity in 2010. On the defense. The Youth Barometer 2010]. (Ed. Myllyniemi S.) Nuorisotutkimusverkosto/Nuorisotutkimusseura, julkaisuja 107 Nuorisoasiain neuvottelukunta, julkaisuja 43; 2010.

[27] McGushin A, Gasparri G, Graef V, Ngendahayo C, Timilsina S, Bustreo F, Costello A (2022). Adolescent wellbeing and climate crisis: adolescents are responding, what about health professionals?. BMJ.

[28] Majeed H, Lee J (2017). The impact of climate change on youth depression and mental health. Lancet Planet Health.

[29] Vornanen R, Törrönen M, Niemelä P (2009). Insecurity of young people: the meaning of insecurity as defined by 13–17-year-old Finns. Young.

[30] Tilastokeskus. Suomen virallinen tilasto (SVT). Esi- ja peruskouluopetus [Preschool and primary school education]. ISSN=1799–3709. Helsinki; 2016. http://www.stat.fi/til/pop/2016/pop_2016_2016-11-14_tie_001_fi.html. Accessed 07 Jan 2023.

[31] Koskelainen M, Sourander A, Kaljonen A (2000). The strengths and difficulties questionnaire among finnish school-aged children and adolescents. Eur Child Adolesc Psychiatry.

[32] Goodman R (1997). The strengths and difficulties questionnaire: a research note. J Child Psychol Psychiatry.

[33] SDQ-info. SDQ; 2017. http://www.sdqinfo.org. Accessed 07 Jan 2023.

[34] Goodman A, Lamping DL, Ploubidis GB (2010). When to use broader internalising and externalising subscales instead of the hypothesised five subscales on the Strengths and Difficulties Questionnaire (SDQ): data from British parents, teachers and children. J Abnorm Child Psychol.

[35] Obel C (2004). The Strengths and Difficulties Questionnaire in the Nordic countries. Eur Child Adolesc Psychiatry.

[36] Youth Research Network & Advisory Council of Youth Affairs. Youth Barometer 2010; 2010. https://www.youthresearch.fi/abstract-youth-barometer-2010. Accessed 12 July 2023.

[37] TENK. Humanistisen, yhteiskuntatieteellisen ja käyttäytymistieteellisen tutkimuksen eettiset periaatteet ja ehdotus eettisen ennakkoarvioinnin järjestämiseksi. [The ethical principles of humanistic, societal and behavioural research with human participants and suggestion for the ethical review]. Tenk, Helsinki; 2009.

[38] Morton SMB, Bandara DK, Robinson EM, Atatoa Carr PE (2012). In the 21st Century, what is an acceptable response rate?. Aust N Z J Public Health.

[39] Hackett L, Theodosiou L, Robson C, Spicer F, Lever R (2011). Understanding the mental health needs of secondary school children in Manchester. Ment Health Fam Med.

[40] Cousins W, Taggart L, Milner S (2010). Looked after or overlooked? An exploratory investigation of the mental health issues of adolescents living in state care in Northern Ireland. Psychol Health Med.

[41] Koskelainen M (2008). The Strengths and Difficulties Questionnaire (SDQ-Fin) among Finnish children and adolescents.

[42] THL. Kouluterveyskysely [School Health Promotion Study]; 2017. https://thl.fi/fi/web/lapset-nuoret-ja-perheet/tutkimustuloksia. Accessed 07 Jan 2023.

[43] Myllyniemi, S. Ed. Monipolvinen hyvinvointi. Nuorisobarometri 2012. [Cross-generational well-being. The Youth Barometer 2012]. Opetus- ja kulttuuriministeriö. Nuorisotutkimusverkosto. Nuorisoasiain neuvottelukunta; 2012. https://tietoanuorista.fi/wp-content/uploads/2013/05/Nuorisobarometri_2012_Verkkojulkaisu.pdf. Accessed 07 Jan 2023.

[44] Johansson A, Brunberg E, Eriksson C (2007). Adolescent girls’ and boys’ perceptions of mental health. J Youth Stud.

[45] Del Giudice M, Wright JD (2015). Gender differences in personality and social behavior. book: international encyclopedia of the social and behavioral sciences.

[46] Bøe T, Hysing M, Skogen JC, Breivik K (2016). The Strengths and Difficulties Questionnaire (SDQ): factor structure and gender equivalence in Norwegian adolescents. PLoS ONE.

[47] Philipp J (2018). Prevalence of emotional and behavioral problems and subthreshold psychiatric disorders in Austrian adolescents and the need for prevention. Soc Psychiatry Psychiatr Epidemiol.

[48] Ryan SM, Jorm AF, Toumbourou JW, Lubman DI (2015). Parent and family factors associated with service use by young people with mental health problems: a systematic review. Early Interv Psychiatry.

[49] Nilsen SA, Breivik K, Wold B, Bøe T (2018). Divorce and family structure in norway: associations with adolescent mental health. J Divorce Remarriage.

[50] Lee AH, Woodruff-Borden J (2018). Roles of emotional reactivity, intolerance of uncertainty, and negative problem orientation on developing childhood worry. Personality Individual Differ.

[51] Gentes EL, Ruscio AM (2011). A meta-analysis of the relation of intolerance of uncertainty to symptoms of generalized anxiety disorder, major depressive disorder, and obsessive–compulsive disorder. Clin Psychol Rev.

[52] Strelau J, Zawadzki B (2011). Fearfulness and anxiety in research on temperament: temperamental traits are related to anxiety disorders. Personality Individual Differ.

[53] Murphey D, Barry M, Vaughn B. Positive mental health: resilience. Child Trends*.* 2013–3. https://www.childtrends.org/wp-content/uploads/2013/03/Child_Trends-2013_11_01_AHH_Resilience.pdf. Accessed 07 Jan 2023.

[54] Stormark KM, Heiervang E, Heimann M, Lundervold A, Gillberg C (2008). Predicting nonresponse bias from teacher ratings of mental health problems in primary school children. J Abnorm Child Psychol.

[55] Lawrence D, Dawson V, Houghton S, Goodsell B, Sawyer MG (2019). Impact of mental disorders on attendance at school. Aust J Educ.

